# Enhanced Signal and Quantitative Detection of Anti-Interferon-Gamma Antibody by Using a Nanometer Biolinker

**DOI:** 10.1371/journal.pone.0160031

**Published:** 2016-07-26

**Authors:** Pei-I Tsai, Adam Shih-Yuan Lee, Shu-Sheng Lee, Ming-Han Chung, Meng-Wei Liu, Chih-Kung Lee

**Affiliations:** 1 Department of Engineering Science & Ocean Engineering, National Taiwan University, Taipei, Taiwan; 2 Department of Chemistry, Tamkang University, New Taipei City, Taiwan; 3 Department of Systems Engineering & Naval Architecture, National Taiwan Ocean University, Keelung, Taiwan; 4 Institute of Applied Mechanics, National Taiwan University, Taipei, Taiwan; University of California Irvine, UNITED STATES

## Abstract

For rapid screening and quantification of an antisera antibody, a nanometer bithiophene-based conductive biolinker can enhanced signal performance and can be used to verify the interaction of an anti-IFN-γ antibody with an IFN-γ protein. The experimental measurements take a generic approach which takes advantage of the functionality of thiophene-based linkers for biosensors. Effects associated with using bithiophene as a biolinker for surface plasmon resonance (SPR) spectroscopy are examined in this paper. By using an atomic force microscope (AFM), it was observed that the morphology of the bithiophene modified gold sensor surface became smoother than the original gold surface. We compared the response and concentration of the anti-IFN-γ antibody on a bithiophene-coated and dextran-coated biochip as well as on different thickness-modified surfaces under SPR relevant conditions. The results indicate that a response to IFN-γ molecules immobilized on a sensor using a bithiophene biolinker improved more than 8-fold when compared to that of a sensor using a dextran biolinker. Furthermore, the regeneration ability of the sensor surface shows good repeatability as only less than a 1% decrease was found after repeating the experimental work over 6 cycles. The characteristics provided us with a good platform for rapid screening, real-time monitoring and quantitative concentration of the autoimmune antibody activities.

## Introduction

For early prevention and treatment of diseases, clinical symptoms with a particular diagnosis and other tests can help to identify a specific disease and identify its mechanism. Quantifying an unusual biomarker in the blood or body fluids is useful for patients suspected of having a disease. There are many types of biomarkers which include proteins, cytokine, chemokine, auto-antibodies, etc. Generally, their concentration varies among the specific biomolecule. Some reported concentration levels include less than 10 pg/mL [1–7], in a few ng/mL [1, 6, 8] range, or within a μg/mL [7, 9] level. For precise and efficient detection, it requires high sensitive enzymes (horseradish peroxidase or alkaline phosohatase) or more expanded amplification to process, detect and analyze the accurate signal of these proteins.

In recent years, much research has been focused on studying the higher risks of immune system protection caused by autoimmune diseases which typically has inflammation as a classical symptom [10, 11]. With continuous research and findings, we now have a better understanding about autoimmune diseases. This has led to great advancement in the detection, evaluation, and confirmation suspected autoimmune illnesses. A typical clinical diagnosis for a positive autoimmune disease diagnosis depends on multiple laboratory tests which may include testing for the presence of inflammatory markers and the presence of autoantibodies [12]. Interferon-gamma (IFN-γ) protein is one of the inflammatory cytokines which usually result from a response in the human body from a direct invader or latent-virus/bacterial infection. Tuberculosis (TB) and other diseases of endocrine systems have also shown similar symptoms and have received similar treatments [10]. In previous works, investigators have shown that functional anti-IFN-γ IgG autoantibodies have a high titer against native human IFN-γ obtained from up to 1/5000 dilution plasma of IFN-γ autoantibody-positive patients. The functional anti-IFN-γ IgG autoantibodies were derived from disseminated nontuberculous mycobacterial infections which blocked the downstream and was inhibitory to early aspects of IFN-γ signal transduction [13, 14]. It is an important mechanism for the immune response from the body's immune system. Enzyme-linked immunosorbent assay (ELISA) is a common laboratory method used to detect and to measure specific biomolecular interactions based on current basic principles and recent clinical applications [11, 13, 15]. It also can screens antisera and determines the antibody titre of each antiserum. The technique studies and recommends optimal specific protein concentration. However, the primary or secondary antibody must be labeled individually to detect and/or to amplify the signal of a bio-interaction within the ELISA. In addition, it cannot provide a real-time response and can thus be time-consuming to implement. Most importantly, it is not a natural interactive response. In accordance with diagnostic evolution, ELISA is typically used to detect analytes which undergo conformation and/or chemistry changes and which are then used to measure, screen and monitor various diseases [16, 17].

On the other hand, previous studies on surface plasmon resonance (SPR) and ellipsometry technology, which detect both refractive index and thickness changes, has been demonstrated to be an important label-free tool for analyzing biomolecular interaction in real-time. In almost all of the biochips used, carboxymethylated dextran was used as the biolinker to modify the sensor surface which provides a flexible antigen/antibody detection platform that is about 100 nm thick [18–22]. Over the years, some advancements for alternative biolinkers within a carboxymethylated dextran matrix framework in a monoclonal antibody immobilization have been developed which makes this family of biolinkers the primary choice for SPR measurements [23].

Spectroscopic ellipsometry (SE), which is an optical technique typically used to measure the thickness of thin films on solid substrates, was tailored to be used for biochip detection when the SPR was being developed. In 1994, Ramsey and Ludema first indicated that the accuracy of thickness measurements of ellipsometry techniques was affected by the roughness of thin films. Their analysis indicated that a 1 nm thickness measurement resolution would deteriorate up to a 10 nm thickness resolution when the substrate roughness increases. This result placed a strong requirement on the roughness of the sensor surface. Combining the above-mentioned finding with the fundamental understanding of both the SPR and SE relies on an electromagnetic (EM) wave which decays exponentially from the sensor surface. This conjecture states that a reduced biolinker thickness can lead to stronger bio-detection signals. This conjecture forms one of the underlying research perspectives for understanding the performance difference between biochips using carboxymethylated dextran matrix and bithiophene as a biolinker.

For rapid screening simulation of an antisera antibody experiment, we synthesized a 1.23 nm bithiophene-based conductive biolinker [24] to enhance and verify the anti-IFN-γ antibody interaction detection capability of our system. In our study, the bithiophene linker was employed to examine the thin film surface coating and its specific binding ability of a sensor surface. Although the traditional coating with ELISA assay has been widely used for decades due to its high sensitivity derived from a secondary antibody induced signal amplification, its limitation is that it often requires a long assay time depending on the reagent used. In addition, an optical density (O.D.) absorbance measurement can only be effectively read after adding an acidic solution to stop the chemical reaction and to stop the darkening of the solution. This can sometimes lead to an erroneous signal measurement. The SPR analysis results obtained on the thiophene-based biosensor surface were compared to that of the ELISA assay approach for the anti-IFN-γ antibody measurements. The experimental data shows that excellent SPR responses were achieved when a bithiophene-coating on the biochip was adopted to immobilize the IFN-γ protein. We looked at the possibility of using a bithiophene-based biosensor surface to quickly screen the anti-IFN-γ antibodies and to assist in the diagnosis of autoimmune diseases.

Our newly developed bithiophene conductive linkers can be used in a variety of different potential applications which can provide us with the potential to further integrate SPR-ellipsometry and electrochemical impedance spectroscopy (EIS) techniques on the same chip and thus expand its application range.

## Materials and Methods

### Simulation of SPR Angle and Sample Thickness

The reflection of different sample thickness on a bithiophene-coated and dextran-coated chip was simulated by FilmWizard Software (v.9.0.4). The simulation parameters were fixed at a 633 nm wavelength light onto a SF2 substrate coated with 50 nm Au and 1 nm Cr. The length of the two type biolinkers were set at 1.23 nm for bithiophene (ChemOffice, PerkinElmer) and at 100 nm for dextran [18, 22, 23].

### Reagents

The anti-human IFN-γ antibody, recombinant human IFN-γ, Mouse IgG horseradish peroxidase conjugated antibody, and monoclonal mouse IgG were purchased from R&D Systems (Minneapolis, MN, USA). The THF (tetrahydrofuran) reagent, phosphate-buffered solution (PBS, 10 mM PBS, 100 mM NaCl, 2 mM KCl, pH 7.4), EDC (N-(3-dimethylaminopropyl)-N’-ethylcarbodiimide hydrochloride), NHS (N-hydroxysuccinimide) and ethanolamine hydrochloric acid (ETA-HCl) were all obtained from Sigma-Aldrich (St. Louis, MO, USA). Tween 20, BSA, TMB (3,3',5,5'-tetramethybezidine), and ELISA plate were obtained from local distributors.

### Self-Assembly of Bithiophene Conductive Linker for Gold Surface Modification

The sensor chips (SIA Kit Au) of plain gold surface were purchased from Biacore (Sweden). We prepared the plain gold surface by using self-modified surface chemistries, i.e., the Biacore’s unique conditions for immobilization were supported by the Biacore local distributor. Our newly developed bithiophene-based biolinkers were attached to the above-mentioned plain gold coated sensor chips in order to assess its ability to bind protein targets. We dissolved the 5 mM 5'-(mercapto)-[2, 2'-bithiophene]-5-carboxylic acid (Formula HO_2_C(C_4_H_2_S)_2_SH with 242 g/mol molecular weight) biolinker with the THF reagent. First, the bithiophene biolinker was synthesized by the team and then self-assembled onto the biochip surface by immersing the chip into the THF reagent for 2 hrs at room temperature [25]. After immersion, the surface of the chips was then chemically modified with a bithiophene biolinker where the functional group formed a carboxylic acid for activation to the attached molecules. The thiophene-based gold chips were rinsed thoroughly with THF, ethanol and distilled water and then dried with nitrogen before being assembled onto the holder. An ellipsometry instrument built by our team was used to measure the physical characteristics of the bithiophene biolinker on a home-made self-coated Au chip [24]. A total of 10 measurement points were taken to obtain the mean values and standard deviation of the bithiophene biolinker, i.e., refraction index (RI) was 1.5355 ± 0.2567 and thickness was 3.0111 ± 0.6964 nm.

### Atomic Force Microscopy

The surface roughness of the Au and Au-bithiophene layer was measured by atomic force microscopy (AFM, NanoScopy, Bruker, USA). The surface roughness values were used to generate a variety of statistical data about the surfaces. The AFM was operated using the mechanical property scanning mode where the samples were imaged in air and the probe was oscillated at a low frequency of 0.376 Hz.

### IFN-γ Protein Immobilization

**Fig 1:**
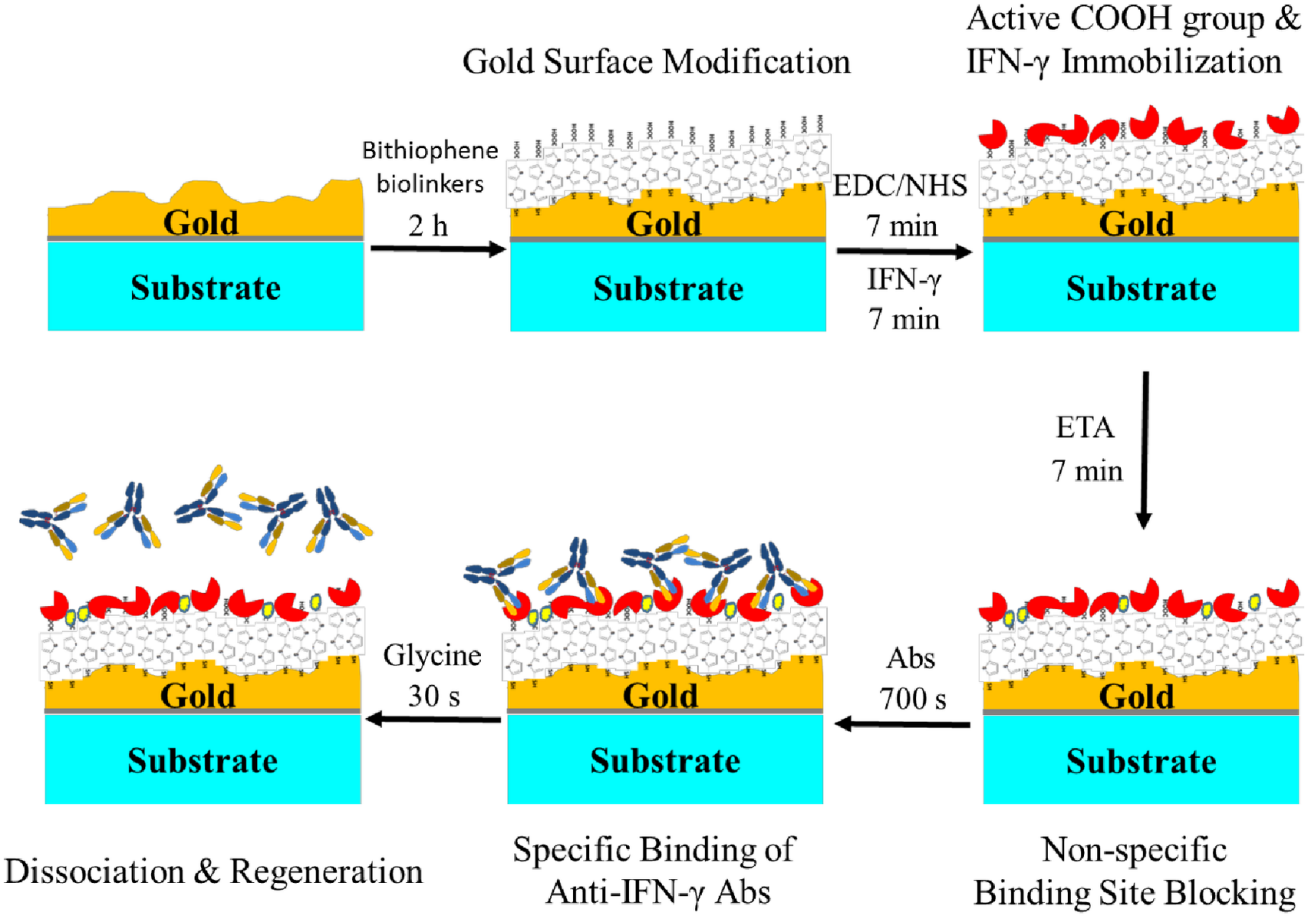
Schematic of bithiophene-based (bithiophene biolinker) SPR-ellipsometry-electrochemical sensor for Anti-IFN-γ antibody detection. The bithiophene biolinker semi-conductive linker was thiolated at the thiophene to achieve self-assembly on the gold surface with immobilized IFN-γ protein.

For biomolecule immobilization, the IFN-γ protein immobilization on thiophene-modified sensor surfaces was monitored using a Biacore T200 instrument while connecting to a continuous flow system. The surface mass by a SPR response unit (RU) was changed directly in real-time. The bithiophene biolinker was activated with 0.4 M EDC and 0.1 M NHS for 7 min at flow rate of 10μL/min. The schematic diagram of the process is shown in Fig 1. The concentration of the immobilized 2 μg/mL IFN-γ protein [26] was defined first by the ELISA assay which was dissolved in PBS (pH 7.4) and used to assess the bithiophene biolinker binding capacity for biomolecular immobilization while operating the Biacore SPR for 7 min at flow rate of 10 μL/min. Finally, we blocked the non-specific binding site of the sensor surfaces by injecting a 1M ETA-HCl solution (pH 8.5) for 7 min. The flow rate of the pumping progress was set at 10μL/min. The non-specific binding of the anti-human IFN-γ antibody was tested before the IFN-γ was immobilized on the sensor for analytical control of the antibody. The surface regeneration was done by injections of 20 mM NaOH. The carboxymethylated dextran chip (CM5, Biacore) was also tested for comparison of non-specific binding as a control for the protein immobilized channel.

### Detection of Anti-IFN-γ Antibody

The anti-human IFN-γ antibody was used as an analyte which served to simulate a human autoimmune antibody and to verify the capability of using a bithiophene-based biosensor for measuring the interaction of IFN-γ and its antibody. The antibody was injected over the sensor chip surface at concentrations of 10-fold, 5-fold and 2-fold serial dilution with the PBS. The specific affinity of the analyte was measured through a 10-fold dilution concentration range. The values obtained with the isotype protein concentration determined by the second channel of Biacore SPR instrument served as the negative control. The affinity and kinetics of the analyte binding to the ligand at various different dilution variant (i.e., different concentration of analyte) were examined using single-cycle kinetics and multiple-cycle kinetics. Using the Biacore T200 evaluation software under a series of regenerated biochip measurement conditions (multiple-cycle kinetics operation), the sensor response at the different analyte concentrations over time demonstrated the association constant *k*_a_ and dissociation constant *k*_d_. Similarly, when we measured SPR responses by gradually increasing the analyte concentration (i.e. in the presence of single-cycle kinetics but with no sensor regeneration) the Biacore T200 results provided us with an equilibrium dissociation constant of *K*_D_ = *k*_d_*/k*_a_. The flow rate of the experimental procedures was set at 30 μL/min. After each measurement, the flow channel was washed with PBS. Isotype antibodies were used in order to identify the IFN-γ immune-negative control at the same concentration present by the introduction of a mouse IgG_2A_ isotype antibody.

### Regeneration

The stability of the immobilized biomolecules anti-IFN-γ antibody 3.33 nM (0.5 μg/mL) on bithiophene-modified surfaces was tested by passing a regeneration solution 10 mM glycine (pH 2.45) for 30 sec at a flow rate of 30 μL/min.

## Results

### Difference of Reflection Rate

**Fig 2:**
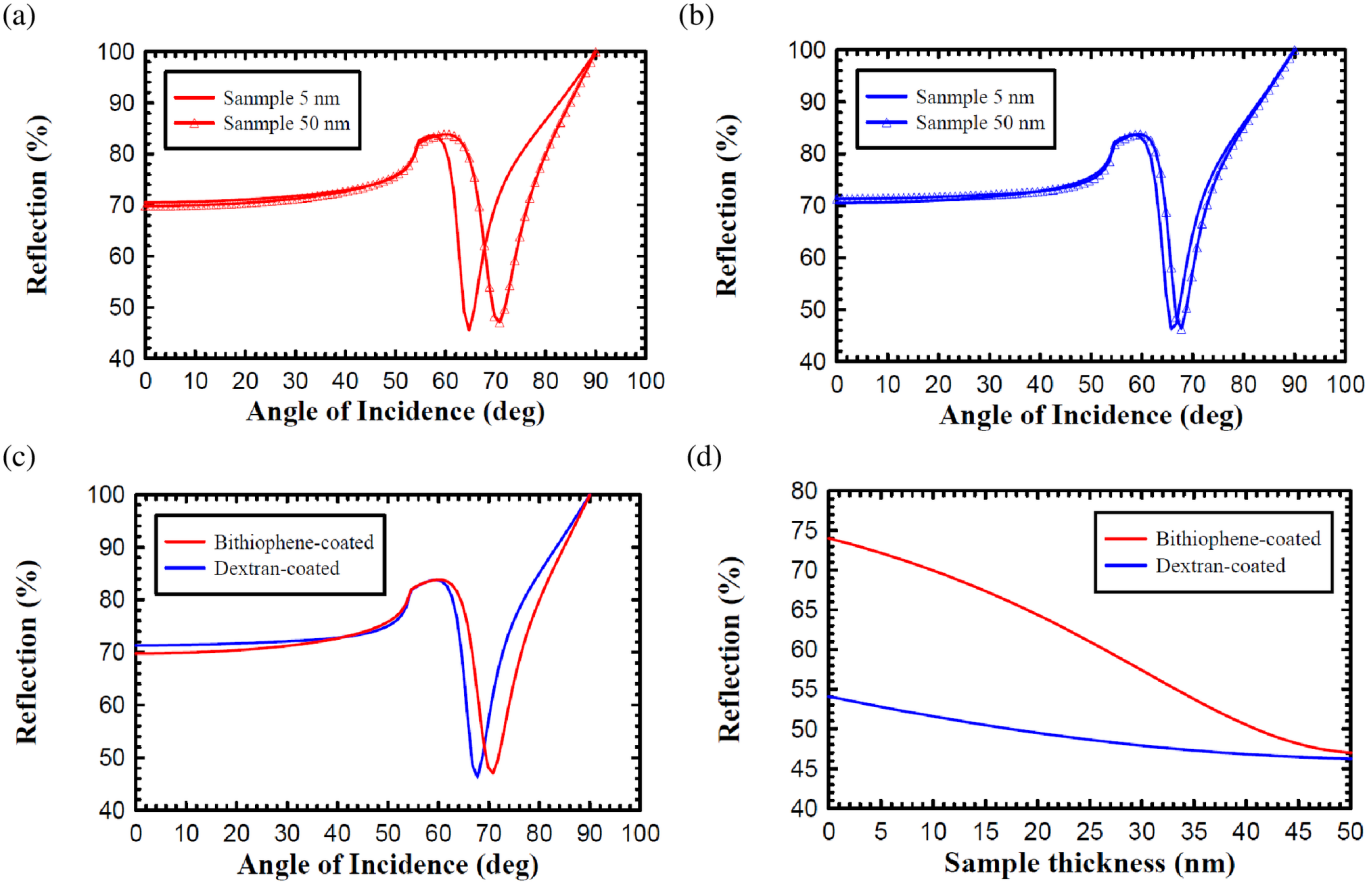
Simulation results of the reflection difference. (a) 6 degree SPR angle shift on the bithiophene-coated chip, (b) 2 degree SPR angle shift on the dextran-coated chip, (c) differences in the SPR incidence angle at 50 nm sample thickness, and (d) reflection differences for the various sample thicknesses.

The simulation results were obtained from the reflection rate difference between the bithiophene-coated and dextran-coated chip at different incident angles and sample thicknesses in Fig 2. There were six degrees shifting of the SPR angle on the bithiophene-coated chip (Fig 2a) which exhibited a 3-fold difference in the incidence angle than that of the dextran-coated chip (Fig 2b) for sample thickness from 5 nm to 50 nm. The SPR angle shows a 70.786 degree angle on the bithiophene-coated chip and a 67.752 degree angle on a dextran-coated chip when the sample thickness was 50 nm (Fig 2c). A reflection rate difference between the bithiophene-coated and dextran-coated reflected light beams was compared to the sample refractive indices at different sample thickness. In Fig 2d, results show an unequal reflected difference dependency on each type of coated chip at various sample thicknesses. With a 15 nm anti-IFN-γ antibody [27] bound to a 5 nm IFN-γ [28] it led to an approximate 20 nm thickness by simply adding the molecule size. The reflection rate difference was 8% on the bithiophene-coated chip and 3.5% on the dextran-coated chip when the sample thickness varied from 5 nm up to 20 nm. It should be noted that the reflection rate of both the chips were similar, i.e., 47% for the bithiophene-coated chip and 46% for the dextran-coated chip, when the sample thickness increases to 50 nm. However, the reflection rate difference for the bithiophene-coated chip was 27% for the sample thickness which varied from 0 to 50 nm. This was a significant increase when compared to the 8% reflection rate difference for the dextran-coated chip in the same condition.

### Morphology of Bithiophene Modified Gold Surface

**Fig 3:**
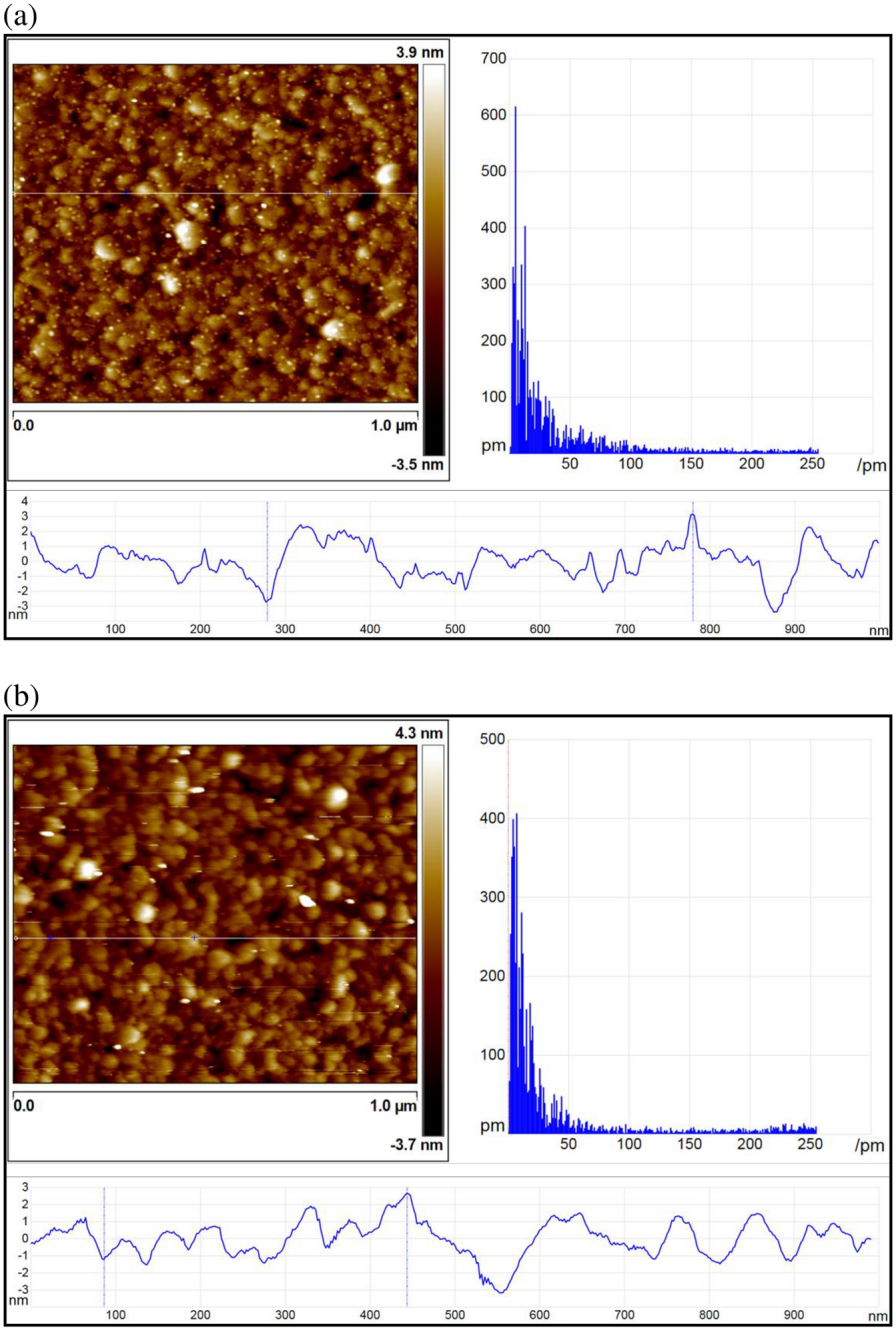
**Topographic AFM images** with (a) A gold surface, Roughness *Rmax* = 5.767 nm, *Rms* = 1.136 nm; *Ra* = 0.979 nm. (b) Modified gold surface with bithiophene biolinker SAM, Roughness *Rmax* = 4.193 nm, *Rms* = 0.775 nm; *Ra* = 0.787 nm. (Scan area = 1μm × 1μm).

Fig 3a shows the topographic AFM images of the gold film surface and Fig 3b shows the Au-bithiophene biolinker modified film surface scanned as seen from an AFM. Various surface roughness parameters such as *Ra* (arithmetic average of absolute values of surface height), *R*_*MS*_ (root mean squared value of surface height), and *Rmax* (maximum peak surface height) were used to characterize the surface roughness from Fig 3 [29]. On the Au-bithiophene biolinker modified surface (Fig 3b), the *Ra* was found to be 0.787 nm. This *Ra* was 20% lower than that of the 0.979 nm bare gold sensor surface roughness (Fig 3a). The root mean square roughness (*Rms*) of the Au-bithiophene biolinker modified surface (Fig 3b) was found to be 0.775 nm and was 30% lower than the 1.136 nm bare gold layer roughness (Fig 3a). In comparison, the *Rmax* was found to be 4.193 nm in an Au-bithiophene biolinker modified surface (Fig 3b) and was also 30% lower than the 5.767 nm bare gold layer roughness (Fig 3a). All of the results shown above indicates that an Au-bithiophene biolinker modified surface was smoother than that of a bare gold layer. This confirms that a bithiophene biolinker will not roughen the bare gold surface and therefore will not interfere with the analyte interactions with the ligand bound on top of the biolinker.

### IFN-γ Protein Immobilization on Bithiophene-modified Gold Surface

**Fig 4:**
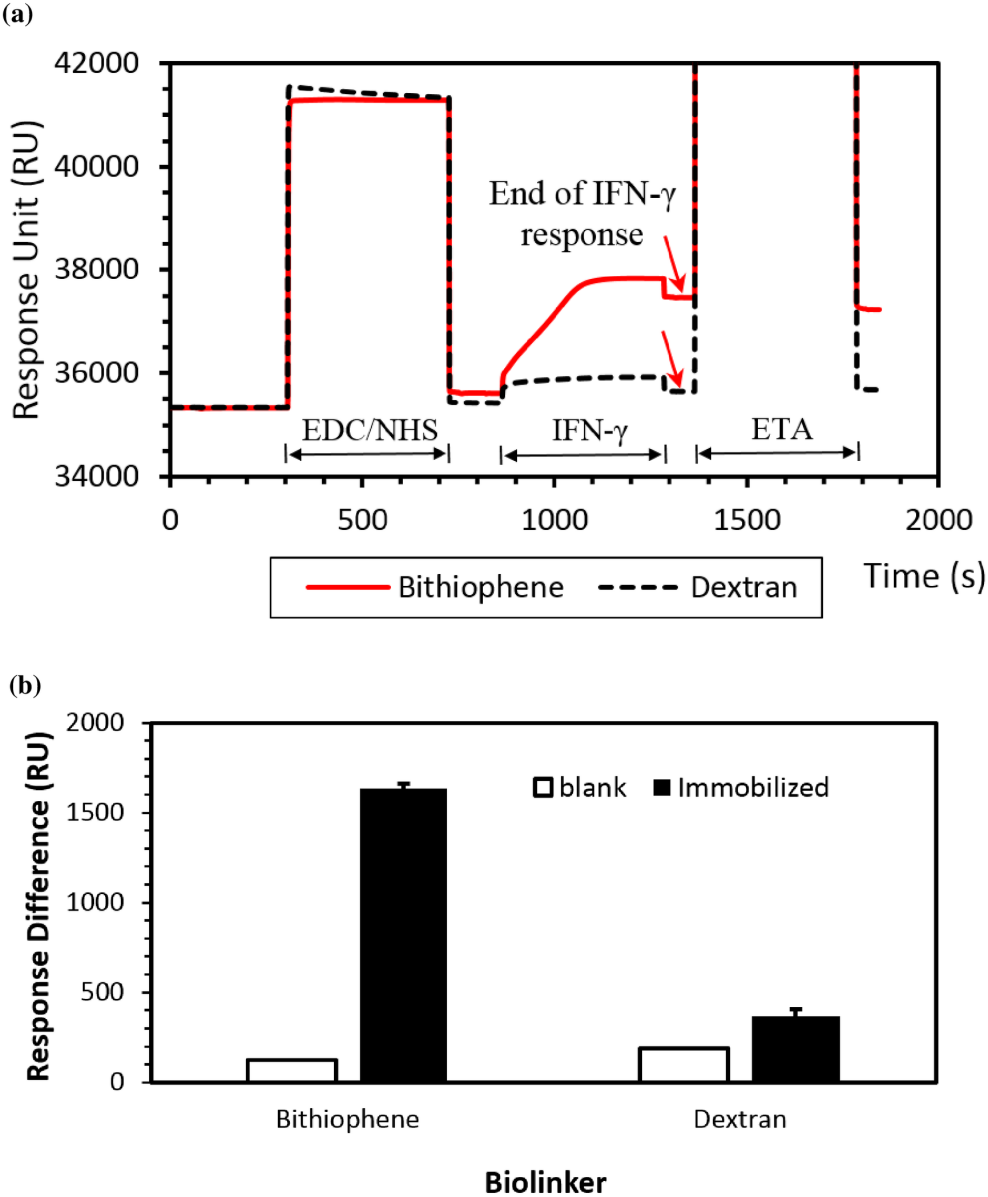
Schematic representation of the IFN-γ attached to the sensor surface. (a) Two real-time sensorgrams resulted from the interaction of the IFN-γ with the bithiophene biolinker- and dextran-coated surfaces of the sensor chip. (b) Comparison of sensor response among the control blank, IFN-γ immobilized on the bithiophene biolinker based biochip, and IFN-γ immobilized on the dextran-coated biochip.

Comparisons of the Biacore SPR responses obtained from the bithiophene biolinker modified sensor chip and the Dextran CM5 biochip are shown in Fig 4. Fig 4a clearly demonstrates the presence of antigens in the immobilization process as indicated by the positive binding response after activation the non-active functional group of the bithiophene biolinker. In addition, a real-time sensor response resulted from the recombinant human IFN-γ protein with the bithiophene biolinker is clearly demonstrated in Fig 4a. An IFN-γ protein immobilized on a bithiophene biolinker sensor chip can be coupled with free amine. Approximately 2000 response units (RU) were obtained when the IFN-γ protein was injected onto the flow cell (Fig 4a). Final measurement of the response difference was a 1633.4±27.98 RU immobilization after injecting the ETA-HCl blocked molecular to bind the free epitope of antigen (Fig 4b). The carboxymethylated dextran chip (CM5, Biacore) were also tested for comparison. The result shows that the response of the IFN-γ molecules immobilized on a bithiophene biolinker-coated sensor is more than 8-fold higher than that of a dextran-coated sensor after subtraction of a response for the blank control (which was obtained by not activating the non-active functional group of the bithiophene or dextran biolinker).

### Interaction of Anti-IFN-γ antibody Bound IFN-γ

**Fig 5:**
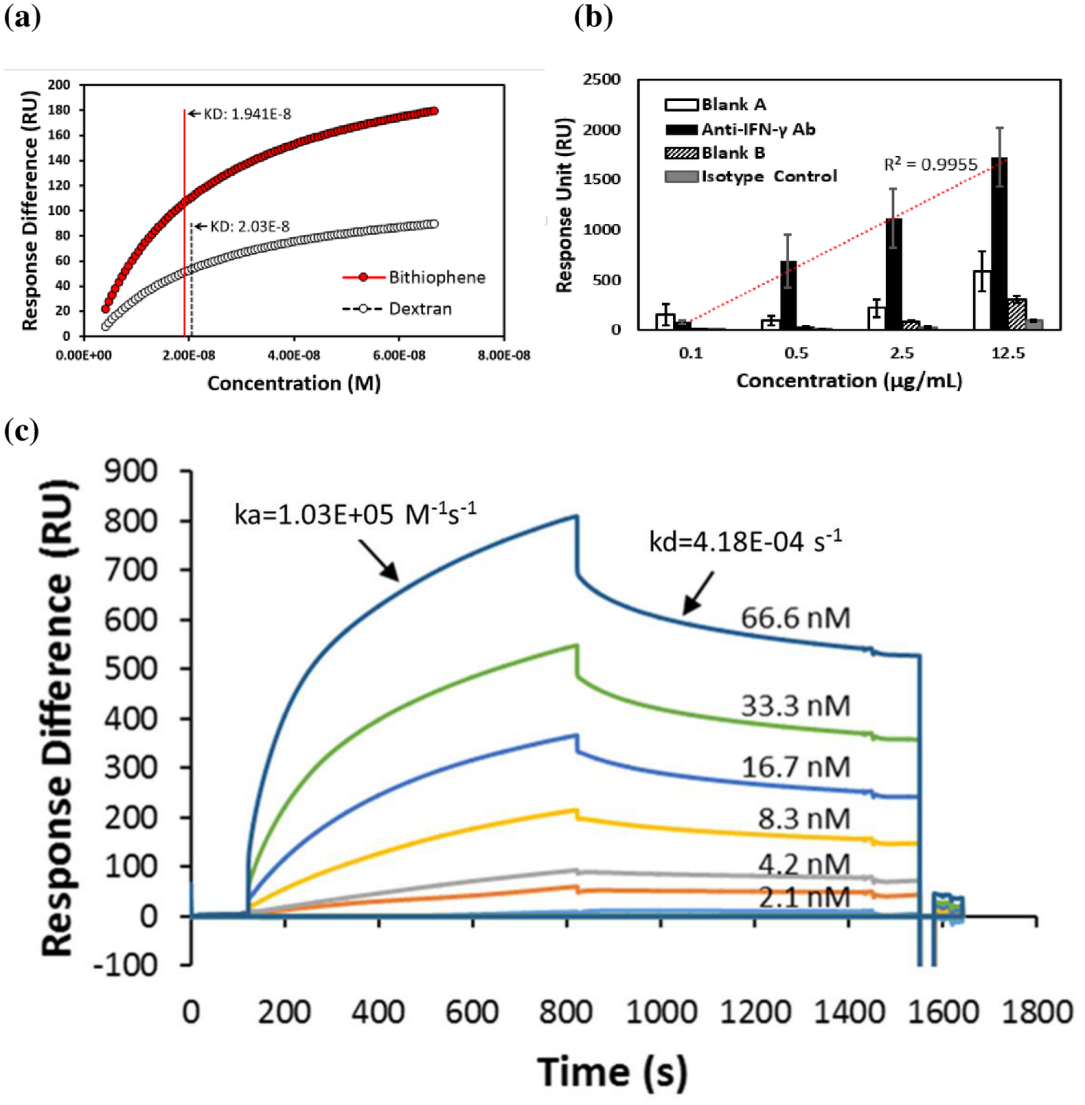
Kinetics and affinity of anti-IFN-γ antibody. (a) Steady state affinity were found to be similar for both sensor. Bithiophene biolinker sensor chip are shown in red solid line. (b) Binding responses of anti-IFN-γ antibody (n = 3) and isotype control (n = 3) with each blank. (c) 1:32 dilution of anti-IFN-γ antibody from 66.6nM to 2.1nM were analyzed. Binding state kinetics shows *ka*: 1.03E+05 M^-1^s^-1^ (SE: 1.6E +03), *kd*: 4.18E-04 s^-1^ (SE: 2.1E-05), K_D_: 4.06E-08 M.

The equilibrium dissociation constants *K*_D_ of anti-IFN-γ antibody and IFN-γ protein as measured from the bithiophene versus dextran biolinker are shown in Fig 5a. More specifically, a specific affinity associated with the bithiophene biolinker was identified to be *K*_D_ = 1.941E-8 and the value for the dextran was *K*_D_ = 2.03E-8. It is worth noting that even though the bithiophene biolinker sensor showed a 2-fold response compared to a dextran-based sensor, the specific affinity of these two sensors are similar. It should be noted that this 2-fold higher sensor response was obtained after the 8-fold immobilization sensor response was demonstrated in the biochip fabricated using the bithiophene biolinker. This increase in sensor response is proof to the advantages of using bithiophene as the biolinker for SPR measurements. After successfully verifying the performance of a bithiophene biolinker-based sensor by comparing its response to a dextran based biochip, experimental data shows that Fig 5b further characterizes the bithiophene-based biolinker sensor In Fig 5b, the analyte antibody concentration from 0.666 nM to 83.25 nM (equals to 0.1 μg/mL to 12.5 μg/mL) was diluted in a 5-fold serial dilution with PBS where the antibodies were injected onto the sensor chip surface with IFN-γ immobilization at various concentrations. The data shows the signal associated with an anti-IFN-γ antibody, a nonspecific mouse IgG_2A_ isotype control and the blank channel with non-IFN-γ immobilization for each analyte. The average of the signal responses of the anti-IFN-γ antibody vs. isotype control were 0.1 μg/mL at 80.0753 RU vs. 6.291, 0.5 μg/mL at 690.6788 RU vs. 14.6254, 2.5 μg/mL at 1118.513 RU vs. 30.6879, and 12.5 μg/mL at 1721.946 RU vs. 95.3279. The obtained coefficient of determination R^2^ of the trend line was 0.9955 for the anti-IFN-γ antibody binding. The isotype control of the IgG_2A_ did not bind to the IFN-γ at a level from 0.1 μg/mL to 2.5 μg/mL. It is clear from Fig 5b which shows the sensor response obtained from measuring the binding of the anti-human IFN-γ antibody to a IFN-γ while injecting 400 μL of 0.625 μg/mL of analyte solution in 10 mM PBS (pH 7.4) is significantly higher than the sensor response simultaneously obtained from the second channel of the Biacore SPR measurement used in the Mouse IgG_2A_ isotype as a negative control. A significantly higher sensor response was also found when compared to that of a blank control. A total of 5 cycles of experimentation were performed to verify the repeatability of the sensors.

In Fig 5c, the samples were analyzed after a 1:32 dilution (66.6 nM of the anti-IFN-γ antibody in a 2-fold serial dilution with PBS to 2.1 nM; 12.5 μg/mL diluted to 0.1 μg/mL) in the above buffer containing anti-human IFN-γ antibody with zero negative control and which were injected with control at the IFN-γ immobilized channels for 700 sec. It took one minute for dissociation and 30 seconds for the sensor surface regeneration by the glycine. The SPR response change was proportional to the amount of ligand binding analyte on the sensor surface. Experimental results have verified the simulated anti-IFN-γ antibody bound IFN-γ interaction detection capability of a bithiophene biolinker sensor. The kinetic association constant *k*_a_ was 1.03E+05 M^-1^s^-1^, where the dissociation constant *k*_d_ was 4.18E-04 s^-1^ and *K*_D_ was 4.06E-08 M at a binding state. Those coefficients were calculated from 2.1 nM to 66.6 nM anti-IFN-γ antibody concentrations. The above-mentioned experimental results suggested that our newly developed bithiophene biolinker conductive linker biosensor can be used potentially to enhance protein interaction application measurements.

In summary, our newly developed bithiophene-based biolinker biochip appears to have better performance when compared to commercially available dextran-based biochips. Furthermore, the difference in specific affinity does not seem to be the main reason for the enhanced signal response. In this work, we theorize that the stronger signals are attributed as a result of a decreased thickness of the bithiophene biolinker.

### Regeneration of Sensor

**Fig 6:**
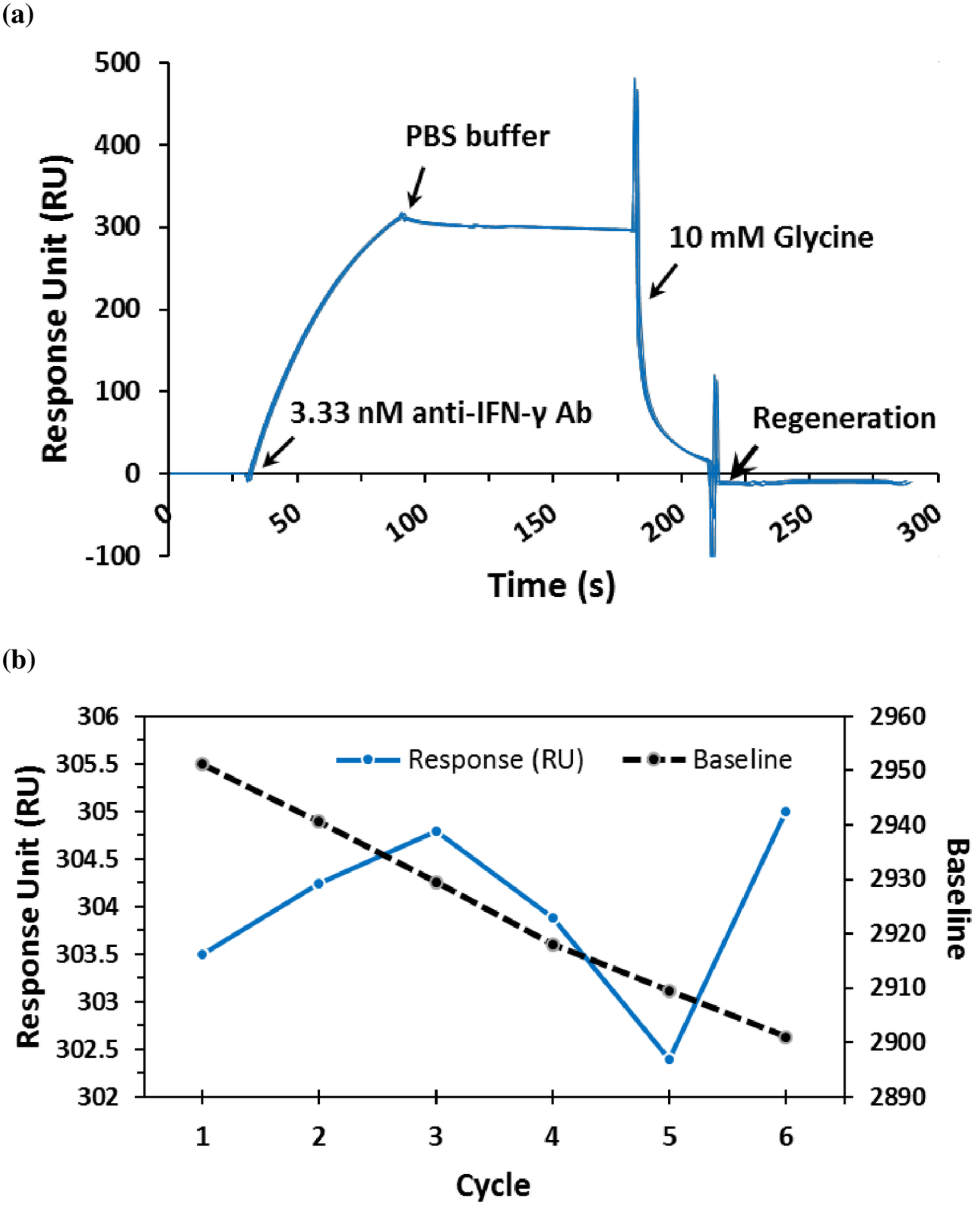
Regeneration from thiophene-modified surfaces after subtraction of the sensor responses of the blank. All experiments repeated 6 cycles under the same conditions. (a) The response line were overlaid from the 6 cycles of the anti-IFN-γ antibody binding and dissociation by 10 mM Glycine (pH 2.45) in real-time monitoring. (b) The response difference from 6 cycles of regeneration on the chip surface.

Fig 6a shows a great repeatable response at an average of 303.97±0.95 RU for 6 cycles of the anti-IFN-γ antibody binding and dissociation by glycine. Its response after a 6 cycle regeneration was a 2.6 RU difference which is less than a 1% decrease after 6 cycles of repeatability (Fig 6b).

## Discussion

In order to detect the interaction between an antigen and the primary antibody on a signal-amplification condition, labeled second antibodies were used throughout various types of assays such as ELISA, western blot and luciferase assay [10, 13, 14]. In this work, we defined a clearer procedure for the experimental protocol for anti-IFN-γ antibody detection which includes using concentrations of IFN-γ immobilization which requires a binding time of the analytes interactions with ligands as well as a robust regeneration condition. With 2 μg/mL IFN-γ, the detection range of the anti-IFN-γ antibody obtained was 0.033 pM to 33.3 pM using an ELISA method (S1 Fig) and from 0.666 nM to 83.25 nM using a biosensor method without resorting to the use of second molecules for signal amplification. This behavior indicates that both the measured technique and the detection range are important for developing viable detection methods for the interactions of anti-IFN-γ antibody and IFN-γ.

More specifically, the expression of native autoimmune antibodies in the human body have been found to be high in concentration. Even though some prior research works have performed diagnostic tests at high concentrations of anti-IFN autoantibodies, no quantitative concentration has been clearly determined. This is probably one of the reasons that the FDA (Food Drug Administration) in the US has not set the standards for anti-IFN antibodies. The newly developed biochip presented appears to have the capability to serve as a reference point to pursue quantitative measurements which can be potentially used to detect anti-IFN-γ autoimmune antibody concentrations within the human body. Furthermore, comparison of the 1/5000 dilution plasma of the anti-IFN-γ autoantibody can be clearly seen in Table 1.

**Table 1:** Comparison of the different analysis methods of the anti-IFN-γ antibody.

*Methods*	*IFN-γ level*	*Anti-IFN-γ autoantibodies/antibody concentrations*	*References*
ELISA	1 μg/well (<, =, > 5 pg/mL)	Qualitative: 1/1000 dilution plasma, presence of anti-IFN-γ antibodies in sera from 10/30 positive of TB patients	Madariaga *et al*., 1998
ELISA	2 μg/mL	Qualitative: 1/1000,000 dilution plasma, a high-titer of anti-IFN-γ antibodies to IFN-γ in the patient’s serum sample	Döffinger *et al*., 2004
ELISA	736 pg/mL	Qualitative: 1/10~1/5000 and above dilution plasma, high-titer anti-IFN-γ antibodies in the plasma of 6/35 patients	Patel *et al*., 2005
Luciferase Immunoprecipitation	N/A	Qualitative: 1/1000 dilution, 85/97(88%) Asian patients had high-titer anti-IFN-γ autoantibodies	Browne *et al*., 2012

In addition to measuring the anti-IFN-γ antibody concentration levels, a bithiophene-based biosensor can also be modified by replacing the antibody or antigen on the sensor chip with another molecule. This replacement can lead to the detection of specific molecule reactivity with other individual biomolecules as well as other antigens in various autoimmune diseases. Compared to an ELISA detection method, a SPR method can be used for detecting the signals of anti-IFN-γ autoantibodies signals directly. Combining the new bithiophene-based biochip with SPR not only leads to a rapid detection measurement but also a robust detection. Various concentration levels can be measured on the same chip for repeated quantitative validation due to the robust regeneration protocol developed (Fig 5c). In addition, the avoidance of secondary antibody can minimize the cross reaction with objects that can lead to a false positive result.

Thereby, the affinity differences of an anti-IFN-γ antibody shown in Fig 5a and 5c are due to a steady state and binding state, whereas *ka* increased in binding state. To further examine the possibility of extending this study to enhance its sensitivity in bio measurements, the effect of a rough surface before and after the thiophene-based measured thickness was studied by using an AFM device. In addition, the influence of the biolinker length on the SPR/ellipsometry detection and the capability of each type of biolinker to bind to the protein of interest must be examined as well.

From commercially available dextran-based sensor chips, the binding capacity of the protein to be detected was tailored by changing the percentage of the carboxylation of the dextran matrix. Regarding the influence of the biolinker length, the length of the biolinker used in dextran CM5 chip (Biacore) was about 100 nm. On the other hand, the length of bithiophene biolinker was calculated to be around 1.23 nm (Chem Office, PerkinElmer), which is similar but not identical to the AFM experimentally measured 3.01 nm length. This 3.01nm length was an average of the values calculated from 10 reference points as measured by the ellipsometer. No matter the average of the numbers, whether it is 3.01 nm or 1.23 nm, both of these measurements are shorter than the actual 100 nm dextran-based biolinker length. Considering that the incident EM wave decayed exponentially on the biochip surface, the shorter the biolinker is, the higher the sensitivity. Nevertheless, the signal detected from the bithiophene-based biochip appears to be higher. This observation suggests that the signal strength has a stronger influence from the biolinker length than that of its protein binding capacity.

## Conclusions

In this study, a bithiophene biolinker was adopted to form a self-assembled monolayer and to immobilize the IFN-γ. The obtained experimental results indicate that integrating this newly developed biochip with SPR correlates well with the various commercially available ELISA products (R&D, USA). Proper protocols were also developed to demonstrate the detection sensitivity, measurement resolution, dynamic detection ranges, and chip regeneration capability of this newly developed biochip. A higher sensitivity on the immobilization of IFN-γ and specific binding of anti-IFN-γ antibodies were both found in the newly developed bithiophene biochips when compared to that of commercially available dextran biochips for SPR measurements. The method presented in this paper demonstrates good performance characteristics include convenient detection and high expression of native autoimmune antibodies for rapid screening and precise quantitative concentration determination. Extending this newly developed biochip to pursue antigen and antibody detection as well as many other bio-detection requirements are discussed and reviewed to better understand the potential of integrating this biochip with SPR or ellipsometry detection measurements.

## Supporting Information

S1 Fig ELISAs record from the anti-human IFN-γ antibody reaction and the detection range of anti-IFN-γ antibody was from 0.033 pM (5.0 pg/mL) to 33.3 pM (5.0 ng/mL). The excitation wavelength was 630 nm.
(DOC)
