# Supplementary material for: Enhanced Signal and Quantitative Detection of Anti-Interferon-Gamma Antibody by Using a Nanometer Biolinker
Source: PLoS One. 2016 Jul 26;11(7):e0160031. doi: 10.1371/journal.pone.0160031 (PMC4961412; doi:10.1371/journal.pone.0160031)
Supplement: S1 Fig — The excitation wavelength was 630 nm. (DOC) [file pone.0160031.s001.doc]

**Supplementary**

Materials and Methods

## Confirmation of Antibody to IFN-γ using ELISA Assay

A common enzyme-linked immunosorbent assay (ELISA) was performed using indirect detection to recognize different concentrations of anti-IFN-γ antibody. In the assay, the antigen were immobilized by direct adsorption to the plate surface. Detection of the antibody was performed using an enzyme-conjugated primary antibody and conjugated secondary antibody (indirect detection). Dilute the antigen with coating buffer (10 mM phosphate-buffered solution, NaCl, Na2HPO4, NaH2PO4, pH 7.4) first. The wells of ELISA plate was coated with the antigen 0.1183 μM (2 μg/mL, recombinant human IFN-γ, R&D Systems) by adding 100 μL of the diluted antigen solution. The completed plate was incubated at room temperature for 2 h and the supernatants were then discarded. Afterwards, the plate was then washed with 400 μL of washing buffer (0.05% Tween 20 in PBS, pH 7.4) for three times. A 200 μL of blocking buffer (1% BSA in PBS, pH 7.4, 0.2 μm filtered) was added to block the non-specific binding sites in the coated wells. The plate was covered with an adhesive plastic and then incubated at room temperature for 2 h. Again, supernatants was discard and the plate was washed with 400 μL of washing buffer for three times. A 100 μL of the diluted antibody samples (anti-human IFN-γ antibody, R&D Systems) was added to wells to capture the antigen. Each concentration of the antibody sample ran in duplicate wells and incubated at room temperature for 2 h. Repeated washing steps. The secondary antibody of mouse IgG horseradish peroxidase conjugated antibody (R&D Systems) was conjugated at 1:1,000 dilution in PBS with 0.1% BSA. The plate was covered with an adhesive plastic and incubate at room temperature for 1h. The plate was washed with 400 μL of Washing Buffer for five times. A 100 μL of TMB (3,3',5,5'-tetramethybezidine) reagent was added before reading the optical absorbance at 630 nm (blue color) immediately in a micro-well reader. Finally, a 50 μL of stop solution (1 N HCl ) was added to each well to stop the color development before reading the optical absorbance at 450 nm (yellow color).

Results

## Confirmation of Antibody to IFN-γ by ELISA Assay

The information from ELISA’s results is provided in S1 Fig that optical density represented the range of antibody concentration in quantifying antigen amount. In ELISAs system, only 2 μg/mL (0.1183 μM) IFN-γ of amount were required to detect anti-IFN-γ antibody. The relative color intensity was significantly higher in the IFN-γ coated group than in the control groups (non-IFN-γ coating). The relative optical density (O.D.) was significantly higher in the IFN-γ coated group than in the control groups. Analytical sensitivity was defined as the lowest measurable concentration which can be distinguished from 5.0 pg/mL concentration of anti-human IFN-γ antibody. Analytical sensitivity range for anti-human IFN-γ antibody determination was from 5.0 pg/mL (0.07 ± 0.012 O.D.) to 5000 pg/mL (2.3 ± 0.029 O.D.) in recognition with secondary antibody conjugated to horseradish peroxidase (HRP) to produce a chemiluminescent colored positive reaction (S1 Fig). As a non-recognized negative control relative to the signal of non-coating IFN-γ well that O.D. averaged to be 0.05±0.0014~0.005.


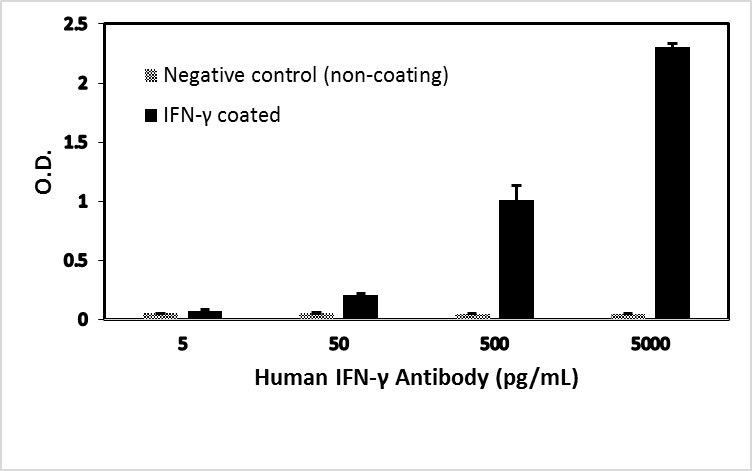


S1 Fig. ELISAs record from the anti-human IFN-γ antibody reaction and the detection range of anti-IFN-γ antibody was from 0.033 pM (5.0 pg/mL) to 33.3 pM (5.0 ng/mL). The excitation wavelength was 630 nm.
